# Mother-male bond, but not paternity, influences male-infant affiliation in wild crested macaques

**DOI:** 10.1007/s00265-016-2116-0

**Published:** 2016-04-28

**Authors:** Daphne Kerhoas, Lars Kulik, Dyah Perwitasari-Farajallah, Muhammad Agil, Antje Engelhardt, Anja Widdig

**Affiliations:** Jr. Research Group of Primate Kin Selection, Max-Planck Institute for Evolutionary Anthropology, Deutscher Platz 6, 04103 Leipzig, Germany; Research Group of Behavioural Ecology, Institute of Biology, Faculty of Bioscience, Pharmacy and Psychology, University of Leipzig, Talstrasse 33, 04103 Leipzig, Germany; Jr. Research Group of Primate Sexual Selection, German Primate Center, Kellnerweg 4, 37077 Goettingen, Germany; Courant Research Centre for the Evolution of Social Behaviour, Georg-August University, Kellnerweg 6, 37077 Goettingen, Germany; Department of Conservation Science, Bristol Zoological Society, Clifton, BS8 3HA Bristol, UK; Primate Research Centre, Bogor Agricultural University, Jl. Lodaya II/5, 16151 Bogor, Indonesia; Faculty of Mathematics and Natural Sciences, Bogor Agricultural University, IPB Darmaga, Jl. Raya Darmaga, 16680 Bogor, Indonesia; Faculty of Veterinary Medicine, Bogor Agricultural University, Kampus IPB Darmaga, Jl. Raya Darmaga, 16680 Bogor, Indonesia

**Keywords:** Male-infant interactions, Paternal care, Paternity uncertainty, Infant survival, Crested macaques

## Abstract

**Abstract:**

In promiscuous primates, interactions between adult males and infants have rarely been investigated. However, recent evidence suggests that male affiliation towards infants has an influence on several aspects of the infants’ life. Furthermore, affiliations may be associated with male reproductive strategy. In this study, we examined which social factors influenced male-infant affiliation initiated by either male or infant, in wild crested macaques (*Macaca nigra*). We combined behavioral data and genetic paternity analysis from 30 infants living in three wild groups in Tangkoko Reserve, Indonesia. Our results indicate that adult males and infants do not interact at random, but rather form preferential associations. The social factors with the highest influence on infant-initiated interactions were male rank and male association with the infant’s mother. While infants initiated affiliations with males more often in the absence of their mothers, adult males initiated more affiliations with infants when their mothers were present. Furthermore, males initiated affiliations more often when they were in the same group at the time the infant was conceived, when they held a high dominance rank, or when they had a close relationship with the mother. Interestingly, paternity did not affect male-infant affiliation despite being highly skewed in this species. Overall, our results suggest that adult males potentially associate with an infant to secure future mating with the mother. Infants are more likely to associate with a male to receive better support, suggesting a strategy to increase the chance of infant survival in a primate society with high infant mortality.

**Significance statement:**

We explore social relationships between males and infants in a promiscuous primate, the wild crested macaque. Our novel approach addresses the nature of affiliations both from males’ and infants’ perspectives. The results show that males and infants form preferential associations. Male-female affiliation, but not paternity, was a significant predictor of interactions initiated both by males and infants. Males initiated more interactions towards infants when the mother was in proximity, while infants initiated more interactions in her absence. Finally, high-ranking males were more likely to initiate interactions towards infants. We demonstrated that paternity is not a good predictor of male-infant affiliations, even in a species with a high reproductive skew and a relatively high confidence of paternity. Our paper is one of the first to show that infants are active agents in establishing and maintaining preferential relationships with males.

**Electronic supplementary material:**

The online version of this article (doi:10.1007/s00265-016-2116-0) contains supplementary material, which is available to authorized users.

## Introduction

The theory of parental investment (Trivers 1972) states that differences between sexes with regard to infant care stem from females investing more time and energy per gamete than males, who instead compete with one another for access to fertile females. Accordingly, adult males (hereafter referred to as males) have been observed to provide infant care in only 10 % of mammalian species (Woodroffe and Vincent 1994). Nevertheless, in the primate genera, paternal care (defined as any behavior a father is directing to his offspring that improves the fitness of the offspring, Trivers 1972) has been observed in up to 40 % of species (Kleiman and Malcolm 1981).

Infant care by male primates is most frequently observed in monogamous pair-bonded species (Whitten 1987), where paternity certainty is high (Griffin et al. 2013). In such settings, fathers maximize their fitness not just by producing a given number of offspring, but by also ensuring their survival (Hamilton 1964). In contrast, a promiscuous mating system often leads to low paternity certainty; thus, father-offspring affiliations are expected to be lower than for monogamous species (Geary 2000) unless fathers are able to recognize their offspring. However, even in promiscuous primate societies, fathers, in some species, have been observed to provide care to a substantial degree, and in doing so increase the probability of their offspring’s survival (“paternal investment” strategy; Smuts and Gubernick 1992) and hence their own fitness.

Paternal care can take different forms. It can entail quite active behavior, e.g., males exchanging affiliative interactions with offspring (rhesus macaques, *Macaca mulatta*, Langos et al. 2013), supporting offspring during agonistic conflict with third parties (savannah baboons, *Papio cynocephalus*, Buchan et al. 2003), or even protecting them against infanticidal males (Hanuman langurs, *Semnopithecus entellus*, Borries et al. 1999). In addition, fathers may have an influence on the fitness of their offspring through paternal effects. For example, sharing spatial proximity with offspring might increase access to food (chacma baboons, Huchard et al. 2013). Furthermore, the mere presence of the father in the infant’s social group may increase the rate of infant maturation (savannah baboons, Charpentier et al. 2008).

In some species, male care can be witnessed in the context of “friendship” (Smuts 1985) between a lactating female and a male that may or may not be the sire. These males form longer-lasting bonds with a mother and may provide care and protection to her offspring (Busse and Hamilton 1981; Anderson 1992; Palombit et al. 1997, 2000). However, a male friend is often a likely sire, i.e., a male present at the time of conception that also mated with the female and hence has some paternity probability (chacma baboons, Moscovice et al. 2009, 2010). An alternative strategy for males to improve their fitness could be to provide care to an unrelated infant. This could increase the male’s future mating opportunities with the infant’s mother in her next reproductive cycle (Kurland and Gaulin 1984; Clutton-Brock 1991; mating-effort hypothesis, Smuts and Gubernick 1992; or care-then-mate strategy, Ménard et al. 2001). Whether this is a widespread strategy in primates remains unclear (van Schaik and Paul 1996). While it has been observed in Barbary macaques (*Macaca sylvanus*, Ménard et al. 2001), no evidence has been found in white-headed langurs (*Trachypithecus leucocephalus*, Zhao and Pan 2006).

While the males’ role and benefits of affiliations with infants has been the focus in previous studies, the infants’ perspective is far less clear. Most studies on male-infant interactions in multi-male groups have, to date, focused on explaining male-initiated affiliative behavior towards infants. In fact, very few studies have investigated the role of infants in affiliative interactions with males, and those that exist are mostly anecdotal (Ransom and Ransom 1971; Guimarães V da and Strier 2001; Moscovice et al. 2009). They do, however, show that infants also take an active part in their relationship with males (Huchard et al. 2013; Langos et al. 2013). Infancy is the most critical period in an individual’s life in terms of survival, as it holds the highest mortality rates in many species (reviewed in Caughley 1966; see also Dunbar 1987; Cheney et al. 2004). Hence, infants can be expected to have evolved survival strategies, for example by forming bonds with their fathers or with males that are friends of their mothers, either to receive protection (Nguyen et al. 2009) or to gain access to richer food patches (Huchard et al. 2013). Under these circumstances, high-ranking males may be preferred social partners for infants, particularly in species in which the risk of infanticide is high (Borries et al. 1999). Clearly, more studies are needed to investigate the infants’ affiliative behavior with males, which males they engage with, and whether this has an influence on offspring survival.

The aim of the present study therefore was to investigate male-infant affiliations in wild crested macaques (*Macaca nigra*) using two analytical approaches in order to take the perspective of both males and infants. Crested macaques are endemic to the island of Sulawesi and live in large multi-male multi-female groups with female philopatry (Duboscq et al. 2013). The social system is highly dynamic due to frequent immigration and emigration of males, leading to frequent changes to the males dominance hierarchy (Neumann et al. 2011) and a short average male group tenure (Marty et al. 2015). Furthermore, paternity is highly skewed towards alpha males due to their successful mate guarding of fertile females (AE unpubl. data). As a result, males fight fiercely for dominance; turn-over in alpha males is particularly high in this species and occur exclusively by males coming from outside the group (Marty et al. 2015). In a recent study, we found evidence that a take-over in alpha male position is the most important determinant of infant mortality in crested macaques (Kerhoas et al. 2014) which suggests that some threat of infanticide may exist even if infanticide has never been directly observed.

In general, infant mortality is high in crested macaques, with 22 % of infants dying or disappearing within their first year despite predator pressure from felids being absent on Sulawesi (Kerhoas et al. 2014). Strategies to improve survival should therefore be particularly important for infant crested macaques. At the same time, males should also be interested in protecting their offspring. So far, it remains unknown for how long fathers stay in the birth group of their offspring, or whether fathers and infants recognize each other. One way for males to assess paternity likelihood may be to use mate guarding and mating success (Buchan et al. 2003). On the one hand, crested macaque females mate promiscuously throughout the majority of the ovarian cycle, increasing the mating success of many males. On the other hand, females are highly monopolized by the dominant male during the fertile phase of their conceptive cycles (AE unpubl. data). Mate guarding would allow assessment of paternity likelihood in the case that males are able to recognize this fertile period.

We propose that for infant crested macaques, it is important to receive protection, particularly against infanticidal males, from high-ranking males, regardless of whether this male is their father or not. We thus predict that infants would actively initiate affiliations with high-ranking males. For males, in contrast, we propose that they should support their own offspring (paternal investment hypothesis) and that high-ranking males would actively provide more care than low-ranking ones based on the reproductive skew observed in this species (AE unpubl. data). Hence, we predict that fathers will provide more affiliation towards their offspring than non-fathers towards non-offspring. Given that males should aim to increase offspring survival, affiliation with fathers should be independent of the mother’s presence in proximity to the infant. At the same time, we test the care-then-mate hypothesis predicting that a male supports unrelated infants in order to create or maintain a social bond with the mother or to advertise its fathering quality so that the mother is more likely to mate with the male in the future. This could be an alternative reproductive strategy for low-ranking males in crested macaques. Consequently, we predict that unrelated males, unlike fathers, interact with infants mainly when mothers are present. Finally, we investigate the effect of mother-male affiliation on infant-male affiliation as they have been found to be linked to one another (Palombit et al. 1997; Moscovice et al. 2009). Accordingly, we predict that the existence of a strong mother-male bond increases affiliations between the infant and the male.

## Methods

### Study population

The study took place in the Tangkoko Reserve (8867 ha) in Sulawesi, Indonesia (1° 31′ 00.1″ N, 125° 10′ 59.9″ E). The research area consists of primary and secondary lowland rainforest (O’Brien and Kinnaird 1997; Rosenbaum et al. 1998; Whitten et al. 2001). Data were collected on three groups of wild crested macaques (R1, R2, and PB), fully habituated to human observers with adults and infants individually identified and continuously monitored by the Macaca Nigra Project. Group sizes varied between 50 to 80 individuals during the study period, with 13 to 25 reproducing females and 4 to 11 adult males in each group.

### Data collection

Behavioral data were collected from October 2008 to September 2010 by DK and four field assistants using a 30-min *focal animal sampling* (Altmann 1974) follow (inter-observer reliability: Cohen’s kappa = 0.67–0.80, significant correlation coefficients between behavioral variables = 0.74–0.96, all *P* < 0.05, Kaufman and Rosenthal 2009). We followed 35 infants (21 males, 14 females) across all three groups. However, our analysis was restricted to a total of 30 infants, as we were not able to sample four infants that died during the course of study, and we excluded another infant with genetic samples for which paternity could not be resolved (see below). Infants were followed from birth to 1 year of age (average weaning age; Kerhoas et al. 2014) excepting five infants who were already born when the study started (1 to 6 months old). During the study period, 30 infants survived to 1 year of age, and five infants died/disappeared during the study. In our study population, we observed that infants spent less than 50 % of their time in proximity of their mother after reaching 5 month of age, suggesting that infants were fairly independent after this time. A total of 3611 h of focal observations were collected with an average of 100.63 h ± 23.87 (SD) per infant that survived to 1 year of age. Observations were conducted from dawn to dusk, and focal sampling was spread evenly throughout the day, sometimes with several focal follows per day per infant.

Simultaneously, we recorded: (i) all social interactions involving the focal animal using continuous focal animal sampling of 30 min duration (Altmann 1974), (ii) proximity of the mother and adult males within a 2.5-m radius of the focal infant using *scan sampling* with 1 min intervals (Altmann 1974), and (iii) all affiliative interactions of the mother and an adult male when both were within a 2.5-m radius of the focal infant as part of focal animal sampling. Since male age was unknown, males were classified as adults if their scrota were fully descended and their canines were fully erupted (Kerhoas et al. 2014). We calculated the proportion of affiliations observed between the mother and an adult male out of the total scans when both were in spatial proximity of the focal infant. All observers were blind to the paternal relationships of the study subjects during behavioral data collection.

We recorded the frequency of agonistic (e.g., half open mouth threat, lunge, chase, etc.; behaviors described for this species in Thierry et al. 2000a) and affiliative interactions. The latter included (1) all socio-positive approaches, that is, no immediate agonistic interaction followed an approach, while the dyad would stay in close spatial proximity of at most 2.5 m for a minimum of 5 s (cf. Langos et al. 2013); (2) social grooming; and (3) friendly behaviors (based on the ethogram described in Thierry et al. 2000a) such as lipsmacks, silent bared teeth face (both greetings), follows (i.e., an individual consistently walks after a moving partner, Thierry et al. 2000a), and peaceful interventions (i.e., intervening in a conflict directing affiliative behaviors towards one of the opponent, Thierry et al. 2000a). Data were recorded using PTab software (PTab Spreadsheet v.3.0; Z4Soft) on Hewlett Packard IPAQ Personal Digital Assistants (model 114) and Psion Workabout Pro M handhelds.

In addition, we collected ad libitum data (Altmann 1974) on male migrations, displacement, or aggressive interactions for the purposes of calculating male and female dominance hierarchies, births and disappearances/deaths of group members, and monthly female sex skin swelling cycles. Female swellings in this species are a fairly reliable indicator of ovulation (Higham et al. 2012), and because females do not present any swelling while gestating, we were able to identify the conceptive cycle without hormone analysis.

### Genetic paternity analysis

Paternity was determined non-invasively by collecting fecal samples before and throughout the study period from all focal infants (*N* = 30), their mothers (*N* = 30), and all potential sires (*N* = 41). Potential sires were defined as all adult males encountered in the study group before or during our study period, irrespective of whether a male was present in the group at the time of a specific infant’s conception (Macaca Nigra Project unpubl. data). We excluded 11 natal subadult males who only reached adulthood during the study, but emigrated shortly after reaching maturation. To store the samples after collection, we used the two-step method (Nsubuga et al. 2004): fresh feces was kept in 90 % ethanol for 24 h and then stored in a tube filled with silica until DNA extraction. We collected and genotyped a minimum of two independent fecal samples for all individuals (except for one infant with only one collected sample) to guard against sample mix-up and animal misidentification. Extractions were done using QIAamp DNA Stool Mini Kit (Qiagen) or GEN-IAL All-Tissue DNA Kit (GEN-IAL GmbH). Samples were genotyped on a total of 12 highly variable microsatellite markers (AE unpubl. data). Products were analyzed with an ABI PRISM3100 automated sequencer and the ABI peak scanner software. We used a combination of the multiple tube approach (Taberlet et al. 1996; Taberlet and Luikart 1999) and the two-step multiplex PCR to increase the accuracy of the results (PCR details in Engelhardt 2004; Arandjelovic et al. 2009). A heterozygous genotype was accepted when both alleles were confirmed at least two times per extract, i.e., a total of four independent PCRs were required for a given individual. A homozygous genotype was assigned when a single allele occurred in six independent PCRs, in order to control for allelic dropout (Taberlet et al. 1996; Taberlet and Luikart 1999; Engelhardt et al. 2006). In case one heterozygous genotype appeared within the six PCRs, we did up to 11 PCR replications to ensure that we reported a true genotype (Taberlet et al. 1996). In our genetic dataset of 176 individuals, the mean observed heterozygosity was 0.77 ± 0.06 (mean ± SD), the mean number of alleles per locus was 6.08 ± 1.78 (mean ± SD), and the mean polymorphic information per allele was 0.66 ± 0.08 (mean ± SD). There was no deviation from the Hardy-Weinberg equilibrium and no evidence of a null allele occurring at these loci (all calculation performed with CERVUS 3.0, Kalinowski et al. 2007). To assess the minimum number of loci required to assign paternity reliably, we calculated the sibling probability of identity (Waits et al. 2001; Schubert et al. 2011). The sibling probability of identity, i.e., the chance of encountering siblings with an identical genotype on a defined number of markers was found to be reasonably low (0.001) when eight loci were used. Thus, we genotyped each individual at a minimum of eight loci, with an average of 11.92 markers ± 0.31 genotyped per individual. Maternity derived from field observations was genetically tested and confirmed for all mother-infant pairs (*N* = 30) in our study which were subsequently used in the paternity analysis. We only determined father-offspring and mother-offspring dyads.

For paternity assignment, we considered all genotyped males as potential sires for all 30 infants. We used a combination of exclusion and likelihood analyses as follows. For 18 infants, all potential males were excluded on at least two loci, with the exception of the assigned sire, who matched the mother-offspring pair at all loci. In eight cases, all potential males were excluded on at least one locus, with the exception of the assigned sire, who matched the mother-offspring pair. In three cases, which were all genotyped on 12 loci, there were no male without mismatch to the mother-offspring pair, but only one male with one mismatch and all the other potential sires with at least two mismatches. In one case, all males were excluded on at least two loci, but two males without a mismatch remained. The assigned sire was the male that was present at the time of conception in the group of the offspring, as the other male was not present in any group at the time and immigrated a year later. All paternity assignments were additionally supported at the 95 % confidence level by the likelihood method calculated by CERVUS 3.0 (Kalinowski et al. 2007).

### Statistical analysis

- Specific male-infant dyads
  To test whether specific male-infant dyads were affiliating at random or displayed more frequent social interactions than expected at random, we performed permutation tests (Adams and Anthony 1996; Edgington and Onghena 2007; Whitehead 2008) on contingency tables containing the frequency of affiliative interactions initiated by infants towards males and vice versa, separately for each study group. We permuted the identities of the initiator of the interactions across the identities of the interactant, including only adult males that were present during the entire study period into this analysis. As the test statistic, we used a chi-square test (Siegel and Castellan 1988) and applied 10,000 permutations into which we included the original data as one permutation. The two-tailed *P* values were determined as the proportion of chi-square values being at least as large as that of the original data. Permutation tests were calculated in R (version 2.14, R Core Team 2012) using a script written by Roger Mundry.
- Factors influencing adult male-infant interactions
  To investigate which factors influence affiliative interactions between males and infants, we ran Generalized Linear Mixed Models (GLMMs) with a binomial error structure and logit link function (Baayen 2008). GLMMs allow to analyze and control various potential confounding variables simultaneously and are suggested as the optimal approach for data including repeated observations of individuals, as they are capable to avoid false positives and erroneous significances and overly narrow standard errors (Schielzeth and Forstmeier 2009; Barr et al. 2013). We ran two models: in the first model the binary response was whether or not an infant initiated an affiliation towards a male on a given day (hereafter: the infant model) and in the second model the binary response variable was whether or not a male initiated an affiliation towards an infant on a given day (hereafter: the male model). The data comprised all possible male-infant dyads based upon the presence of males and infants per day in a given group. In other words, the response variable was a binary variable: whether the male-infant dyads within a group were observed affiliating or not on a given day. In both models, we included the following predictor variables which were determined on a daily basis.
  *Mother and male rank:* Male dominance rank may have a strong influence on male-infant interactions as observed in other cercopithecine species (e.g., Japanese macaques, Itani 1959; yellow baboons, *P. cynocephalus*, Stein 1984), and mountain gorillas (*Gorilla beringei beringei*, Rosenbaum et al. 2015), because they may offer greater status benefits. An adult male’s interest in an infant may be influenced by the mother’s rank as high-ranking females tend to have a higher infant survival than low-ranking females in some species (e.g., Majolo et al. 2012). However, female crested macaques express a tolerant social style, characterized by low intensity, frequently bidirectional, aggressive interactions, and reconciled conflicts (Duboscq et al. 2013). A previous study found that maternal rank predicted fetal, but not infant survival, suggesting that being a dominant female may, in fact, not increase infant survival in this population (Kerhoas et al. 2014).
  To obtain a continuous measure of adult male and adult female dominance rank, separately (as males usually outrank all females), we used Elo ratings (Albers and de Vries 2001; Neumann et al. 2011) based on displacements and aggressive dyadic interactions observed ad libitum (Altmann 1974) between adult group members. For each observational day, we standardized the Elo rating scores of all adults of the same sex to a range from 0 to 1, in order to obtain comparable ratings across the entire study period. As focal infants are expected to occupy a rank based on the rank of their mother when they reach 1 year of age (Cheney 1977; Datta 1988), we used the mother’s rank as a proxy of the expected infant rank in this study.
  *Mother presence:* In the first year of an infant’s life, mothers are expected to have a large impact on the presence of conspecifics who they tolerate in close spatial proximity of their infant. In addition, the “mate-then-care” strategy predicts that males would be more likely to affiliate with infants when the mother is present. Hence, we included the daily number of scans when the mother was in spatial proximity (i.e., within 2.5 m) of her infant.
  *Mother-Male affiliation:* Friendship is defined as a close, affiliative relationship (Silk 2002) that entails high rate of spatial proximity and grooming (Smuts 1985). In rhesus macaques, male-female friendship has been observed during the birth season (Chapais 1983; Manson 1994), and in Assamese macaque (*Macaca assamensis*), the relationships were found to last at least 2–3 years (Ostner et al. 2013). To investigate the influence of mother-male affiliation (or mother-male friendship) on the probability of adult male-infant affiliation, we calculated the frequency of friendly behaviors (e.g., mainly social grooming but also lipsmack, silent bared teeth face, etc.; hereafter affiliations) the mother and an adult male exchanged when both were in spatial proximity to her infant (i.e., within 2.5 m) during the infant’s focal follows. This predictor was included, as it was found that males both associate and provide care to infants of their female friends (Smuts 1985; Nguyen et al. 2009; Moscovice et al. 2010; Ostner et al. 2013).
  *Paternity:* We identified 30 father-infant dyads out of a total of 224 adult male-infant dyads. Given the high infant mortality in crested macaques, we expected fathers to invest in affiliative relationships to ensure offspring survival. Hence, we investigated if males affiliate more with their own offspring compared to non-offspring although there are more opportunities for interactions between adult males with unrelated infants. In addition, we investigated whether infants affiliate preferentially with their father rather than non-fathers. We omitted focal follows of the infant who did not have its father anymore in the group at the date of the focal (i.e., for the male and infant models).
  *Male presence at conception:* Adult males may establish a relationship with the respective infant based on their presence during the infant’s conception. Given that females mate with multiple males during their likely conception, these males are also likely to have fathered the infant (Borries et al. 1999; Moscovice et al. 2010). We considered all adult males that were present in the group during the conceptive cycle of a given mother per focal infant. We included this variable in the male model only, as infants (not yet born) cannot have witnessed which adult males were present or not at conception.

In addition, we expected several variables to interact with the presence of the mother. In fact, the opportunity of infants to interact with an adult male could be highly dependent on the mother presence and behavior which could either restrict or promote infants’ affiliations with a given male, depending upon the male’s rank or paternity probability (reviewed in Widdig 2007). Thus, we included the three-way interaction between paternity, male rank, and mother presence. To investigate the influence of mother-male friendship on the male-infant affiliation, we also included the three-way interaction between paternity, male rank, and mother-male affiliation. We also included the five associated lower level two-way interactions into the infant and male model.

In addition to these test predictor variables, we controlled for the following variables known to have an effect; however, they are not interpreted (cf. Mundry 2014). As in other female phylopatric species, infants may develop their relationships with adults differently according to their sex (Bolin 1981; Evans et al. 2012; Langos et al. 2013). Hence, we controlled for *infant sex*. To account for the social development taking place in the first year of life, when infants are not yet weaned, we included also the daily *infant age. Number of adult males in the group* may influence male-male competition and therefore the frequency of male-infant interactions. The activity required to fulfill energetic needs such as foraging may also constrain the time males spent affiliating with infant. Therefore, we controlled for the variation of fruit availability by including *seasonality* as the cosine and sine of date (i.e., day within year) into the model (Stolwijk et al. 1999). As rainfall seasonality affects infant survival in this population (Kerhoas et al. 2014), seasonality may also have an influence on the occurrence of male-infant interactions. *Male tenure* (i.e., the number of days an adult male spent in a group) was added as a variable because this should influence the degree of familiarity between adult males and mothers, which might be important for the formation of friendships. In fact, the duration of male residency in a group is very variable in this species (mean ± SD = 766.5 ± 396.7 days, range = 1 to 1549 days). Finally, the *observation time* (i.e., number of scans recorded by day) was incorporated in the model as an offset variable, to account for observation efforts (McCullagh and Nelder 1989). Random effects included in the model were the identity of the *adult male*, the *infant*, the *dyad* (i.e., adult male and infant identity combined), the *social group*, and the *day* of data collection (Table S1 in the Online Resource shows all factors included in the infant and male model). Moreover, to account for random variation in the mean response and the strength of the effect of predictors among different individuals or dyads, as well as to keep type I error rate at 5 %, we included all possible random slopes (15 in total, see details in Online Resource) for the tested variables and interactions (Barr et al. 2013).

Furthermore, our datasets were likely to show temporal autocorrelation as the response (organized in the succession of observational days) may not be independent (Burnham and Anderson 2002). Thus, we included two autocorrelation terms (AC terms), one for the infant and one for the adult male, in each models (as in Langos et al. 2013). The AC terms were calculated by first deriving the residuals from the model described above. Then, separately for each data point, we averaged the residuals of all other data points. The contribution of the residuals was weighted by a function of the time lag between them and the specific data point. These weighted functions had the shape of a Gaussian distribution, and their standard deviations were determined such that the likelihood of the model with the autocorrelation was maximized. The AC terms were calculated using a function written by Roger Mundry.

All statistical analyses were carried out in R (version 2.14, R Core Team 2012). Prior to running each model, we checked all predictors for their distribution and, as a consequence, log-transformed mothers’ presence to achieve a more symmetrical distribution. Then, we z-transformed all continuous predictors to a mean of zero and a standard deviation of one to get comparable estimates and an easier interpretable model with regard to the interactions. The GLMM was fitted using the function “lmer” of the lme4 package (Bates et al. 2011). We used a likelihood ratio test (LRT, R function “anova”, Dobson 2002) to compare the fit of the full model with the fit of the null model (including only the AC terms, offset, control, and random factors) to determine whether the predictor variables as a whole influenced the response variable (Forstmeier and Schielzeth 2011). Given the significance of the full model, we tested the significance of interaction also with LRT’s comparing the full model with a model reduced by the respective interaction. In the case an interaction was non-significant, we removed it from the model to reliably infer about the respective lower terms it included. To check for the assumptions of our models, we calculated Variance Inflation Factors (VIF, Quinn and Keough 2002) using the function “vif” of the R package “car” (Fox and Weisberg 2011). Given that the largest VIF for both models reached 3.35, we concluded that collinearity was not a severe issue. We assessed model stability by comparing the estimates derived by a model based on all data with those obtained from models with the levels of the random effects excluded one at a time, which indicated that no influential cases existed.

## Results

We observed a total of 24,082 affiliations between infants and males distributed over 449 days out of a total of 465 days of observations (96.5 %). Furthermore, we found that 22.5 % ± 4.6 (mean ± SD) of all interactions of focal infants involved an adult male; 81.5 % ± 8.4 of all male-infant interactions were affiliative, the remaining being aggressive interaction. Infants initiated a total of 15,290 (63.5 %) male-infant affiliations (mean of 509 ± 158 per infants). The strength of male-infant association varied between dyads with a mean rate of infant affiliations towards males of 0.043 ± 0.054 (mean ± SD) and a mean rate of male affiliations towards infants of 0.019 ± 0.016 (mean ± SD). The vast majority of affiliations initiated either by infants or adult males consisted of tolerated approaches (infant 91.6 % ± 3.1, male 96.4 % ± 11.8 of all their interactions initiated). In addition, infants’ second and third most frequently initiated behaviors towards males were following (3 % ± 2.1) and lipsmacks (2.9 % ±2.6; Thierry et al. 2000a). In contrast, the behaviors that adult males initiated most, after tolerated approaches, was silent bared teeth faces towards infants (1.5 % ± 6.0) and peaceful interventions (0.4 % ± 0.7). Infants groomed by adult males were rarely observed (0.1 %). The majority of fathers (86.7 %) were still present when the offspring completed their first year of life. Finally, we calculated the average number of scans an infant was in proximity of a male and its mother. When an infant was near a male, the mean number of scans where the mother was also present was 230 ± 96, while, on average, in 1123 scans ± 427 the mother was absent. This was a five times higher probability that an infant was near a male without its mother. When an infant was near the mother, the mean number of scans a male was also present was 220 ± 95, and in 1380 scans ± 463, on average, no males were present, representing a six times higher probability of infants being only with the mother.

### Specific male-infant dyads

The results of the permutation tests showed that adult male-infant dyads did not affiliate at random, regardless of the group considered and whether infants or adult males initiated the interaction (Table 1). Hence, infants and adult males, respectively, had preferred affiliative interaction partners. More than half of the males (53 %) had several preferred infant partners (two or three preferred infants). The majority of infants (26 out of 30, 86 %) approached only one preferred male partner. Finally, 29 % of males (10 out of 35 males) did not preferentially affiliate with any infants, whereas only 3 % of infants (1 out of 30 infants) had no male associates.

**Table 1:** Results of the permutation tests for both infant- and male-initiated affiliations for each study group. Chi-square values are calculated from observed values

Initiator	Recipient	Group	Total N	Chi-square	*p* value
Male	Infant	PB	2651	250.4	<0.001
Infant	Male	PB	5817	448.2	<0.001
Male	Infant	R1	3113	617.2	<0.001
Infant	Male	R1	4231	1370.2	<0.001
Male	Infant	R2	4382	873.4	<0.001
Infant	Male	R2	6478	1957.9	<0.001

### Factors influencing adult male-infant affiliations

**Fig. 1:**
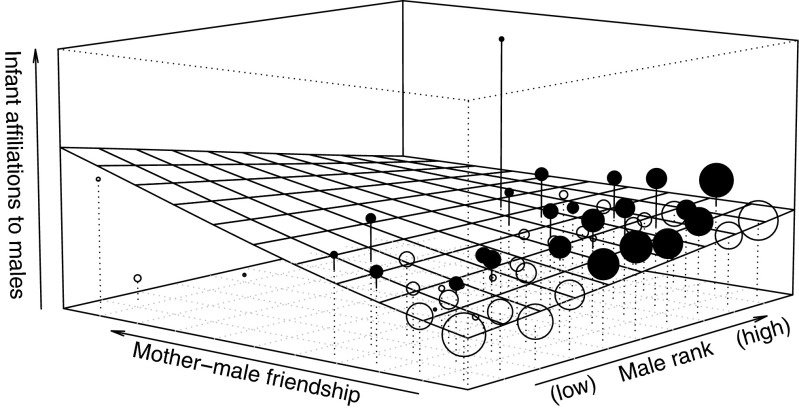
Influence of the interaction between male dominance rank and the strength of mother-male friendship on the probability of affiliation of an infant towards a male. The *plane* depicts the predicted values calculated by the GLMM with each grid representing the mean value per square of the predicted mixed model. *Circles* represent empirical mean affiliation value per square and the size of the circles is proportional to the number of data points. The *filled circles* depict the data points that exceed the estimated value, and the *open circles* depict the data points that fall below the estimated values

- Infant model
  When comparing the fit of the full model with the fit of the null model (i.e., a model including only the control, offset, and random variables and the AC terms), the infant model was significantly different to the null model. Hence, the set of predictors tested had a clear influence on the probability of infants’ initiated affiliations towards adult males (LRT: **χ**^2^ = 26.15, df = 13, *P* = 0.016). To achieve the final model, all interactions were tested for their significant contribution to the full model using a LRT. This resulted in the removal of the two three-way interactions male rank*paternity*mother presence (**χ**^2^ = 1.32, df = 1, *P* = 0.25) and male rank*paternity*mother-male affiliation (**χ**^2^ = 0, df = 1, *P* = 0.97). We also removed the following two-way interactions from the infant model: paternity*mother presence (**χ**^2^ > 0.17, df = 1, *P* = 0.68), male rank*mother presence (**χ**^2^ = 0.01, df = 1, *P* = 0.93), male rank*paternity (**χ**^2^ = 0.63, df = 1, *P* = 0.42), paternity*mother-male affiliation (**χ**^2^ = 0.12, df = 1, *P* = 0.72). We kept the two-way interaction between male rank and mother-male affiliation in the final infant model as it had a significant influence on infant affiliations towards males (**χ**^2^ = 4.36, df = 1, *P* = 0.037). The results of the interaction showed that infants initiated affiliations predominantly towards high-ranking males, but this effect seemed to be modulated by the affiliative bond between the mother and the respective male, whereby if a male had a stronger relationship to the mother, the infant-initiated interactions more towards this male even if it was low ranking (Fig. 1). In addition, infants initiated interactions significantly more towards adult males when the mother was not present in proximity (Table 2). Interestingly, infant affiliation was not influenced by mothers association with the infant’s sire as the two-way interaction between paternity and mother-male affiliation was not significant. Finally, neither paternity nor mother’s rank had a significant effect on infants initiating affiliations towards males (Table 2).

**Table 2:** Results of GLMM analyses of the infant model: the significant factors influencing the affiliations of infants toward males are marked in bold (values not shown for the variables comprised by a significant higher interaction and for the control variables, which can be found in the Online Resource)

Fixed effects:	Estimate	SE	LRT df	LRT **χ** ^2^	*P* of LRT
Intercept	−5.32	0.20			
Infant AC term	0.40	0.02	1	442.66	<0.001
Male AC term	0.23	0.02	1	140.53	<0.001
Paternity	0.13	0.12	1	1.11	0.292
Mother rank^a^	0.03	0.02	1	1.56	0.212
Mother presence	−0.17	0.05	**1**	**5.27**	**0.022**
Male rank* Mother-male affiliation	−0.07	0.03	**1**	**4.36**	**0.037**

^a^Larger values indicate larger rank
- Male model
  Again, the set of predictors tested had a clear influence on the probability of adult males’ initiation of affiliations towards infants (LRT, comparing the fit of the full model with the fit of the null model: **χ**^2^ = 26.34, df = 14, *P* = 0.023). To achieve the final model, we tested all interactions and removed the two three-way interactions male rank*paternity*mother presence (**χ**^2^ = 0.17, df = 1, *P* = 0.68) and male rank*paternity*mother-male affiliation (**χ**^2^ = 0.07, df = 1, *P* = 0.79). We also removed all the two-way interactions from the male model: paternity*mother presence (**χ**^2^ = 1.98, df = 1, *P* = 0.15), male rank*mother presence (***χ***^2^ = 0.92, df = 1, *P* = 0.33), male rank*paternity (**χ**^2^ = 2.15, df = 1, *P* = 0.14), paternity*mother-male affiliation (**χ**^2^ = 0.80, df = 1, *P* = 0.37), male rank*mother-male affiliation (**χ**^2^ = 2.12, df = 1, *P* = 0.14).
  With no three- or two-way interactions significant, all factors were solely tested as single effects, whereby each single effect is controlled by the effect of all others. The results of the final male model showed that adult males that were present during the infant’s conception period were significantly more likely to initiate affiliation with the infant than males absent at conception (Fig. 2). In addition, high-ranking males tended to be more likely to initiate affiliations with infants than low-ranking males (Table 3). A mother’s proximity to her infant significantly increased the probability of an adult male’s affiliation with her infant (Fig. 3); in other words, males interacted more with an infant when the mother was present. Furthermore, mother-male affiliation also increased the probability of male affiliations towards infants. As in the infant model, paternity and mother rank was found to have no significant influence on the probability of adult males initiating affiliation towards infants.

**Fig. 2:**
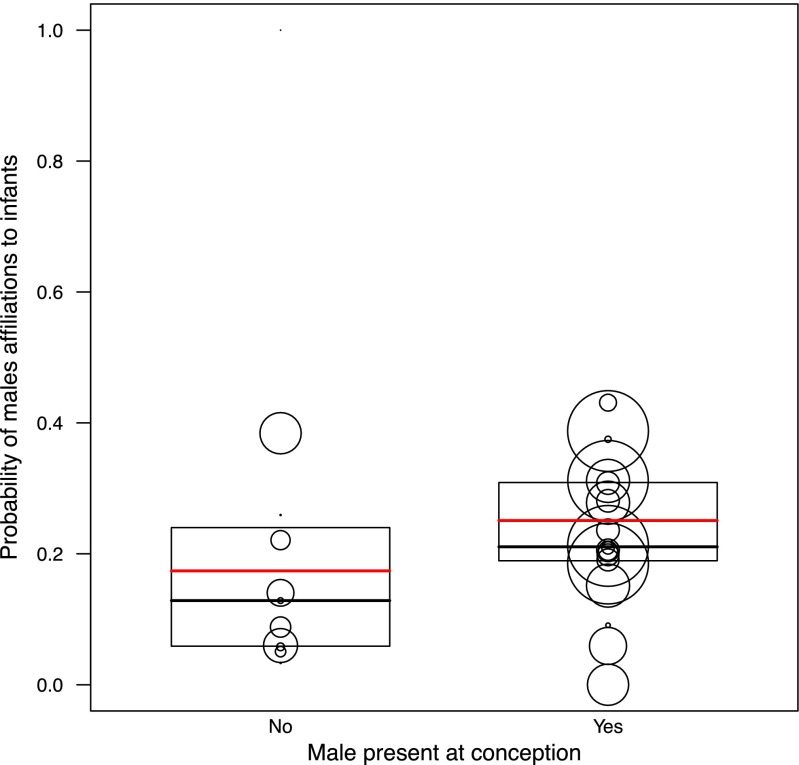
Influence of male presence at infant conception on the probability of males to affiliate with an infant. The *boxes* represent the first to the third quartile of observed values, *solid vertical lines in the boxes* show the median, and *red vertical lines in the boxes* show the values fitted by the model. The *size of the circles* is proportional to the number of male-infant dyads observed

**Table 3:** Results of GLMM analyses of the male model: the significant factors influencing the affiliations of males towards infants are marked in bold (values not shown for the control variables which can be found in the Online Resource)

Fixed effects:	Estimate	SE	LRT df	LRT **χ** ^2^	P of LRT
Intercept	−5.47	0.18			
Infant AC term	0.18	0.02	1	89.27	<0.001
Male AC term	0.19	0.02	1	90.01	<0.001
Paternity	−0.08	0.10	1	0.53	0.465
Mother presence	0.10	0.03	**1**	**4.43**	**0.035**
Male presence at conception	0.35	0.14	**1**	**6.02**	**0.014**
Male rank^a^	0.14	0.06	**1**	**3.15**	**0.015**
Mother rank^a^	−0.01	0.02	1	0.15	0.696
Mother-male affiliation	0.07	0.03	**1**	**5.01**	**0.025**

^a^Larger values indicate larger rank

**Fig. 3:**
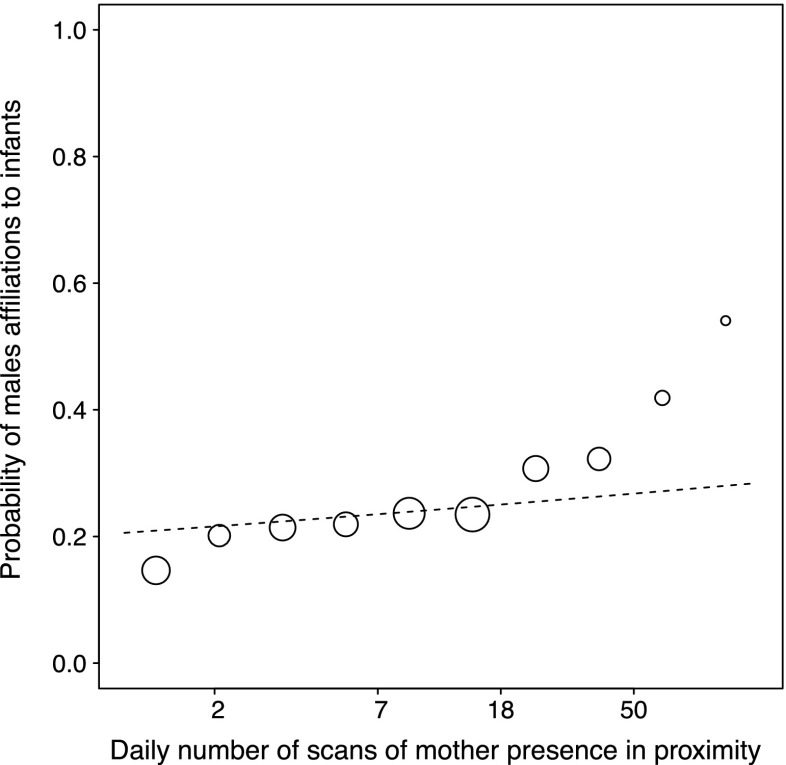
Influence of mother spatial presence on the probability of males to affiliate with her infant. The x-axis is a logarithmic scale of mother spatial presence per day per dyad. The *dotted line* shows the predicted value calculated by the model. The *size of the circles* is proportional to the number of male-infant dyads observed

## Discussion

The results of our study suggest that, in wild Sulawesi crested macaques, adult male-infant interactions are mainly positive, with specific males and infants forming affiliative bonds. Adult male-infant affiliations were twice as common when compared to rhesus macaques (Langos et al. 2013) and, interestingly, infants initiated two thirds of them. Paternity did not significantly affect interactions initiated by adult males or infants; therefore our data do not provide any support for the paternal investment hypothesis. However, the probability of male-infant affiliation was significantly higher if the male was present at the infant’s conception, a cue that males may use to assess their paternity likelihood, unless they use other cues such as mating success (cf. Buchan et al. 2003), which we were unable to include here. Male dominance rank also influenced their likelihood to affiliate with infants, as higher-ranking males interacted more with infants than lower-ranking males. Interestingly, a male’s affiliation with the mother and mother’s presence respectively were both important factors determining males’ affiliations with infants, which may be an indirect indication to crested macaque males possibly using the “care-then-mate” strategy. At the same time, our results clearly show that male-infant interactions are predominantly instigated by infants and thus obviously in their interest. We found a significant two-way interaction between male rank and mother-male affiliation on the probability of infants associating with adult males suggesting that infants preferred to affiliate with high-ranking males or low-ranking friends of their mother. In most behavioral studies investigating adult male-infant interactions, the data collection focuses on adult males, thus exploring the underlying evolutionary mechanisms for male care. However, our results clearly show that male-infant interactions are predominantly instigated by infants, in accordance with previous studies (Moscovice et al. 2009; Huchard et al. 2013, but see Langos et al. 2013). Infancy is the life stage that holds the highest mortality (Dittus 1979; Blomquist 2013), so it is reasonable that strategies favoring survival would be strongly selected for. In fact, we have observed that infants target one specific male, increasing the chance of developing a bond with a potential protector, which may in turn promote infants’ chance of survival. The high initiation rate of infants may be linked to the high infant mortality rate as male-infant interactions are in fact quite common in our population. In chacma baboons, a species suffering a very high infant mortality (Palombit 2000), infants or juveniles have been observed to mainly initiate proximity with males (Moscovice et al. 2009; Huchard et al. 2013). Hence, future studies should investigate whether male-infant interactions are more likely to be initiated by infants, not males, when infant mortality is high, and whether there is an influence on infant fitness and survival.

### Infant model

Infants in our study initiated affiliations preferably towards high-ranking males, but this effect depended on the intensity of mother-male affiliation: with increasing mother-male bond, infants showed a preference even for low-ranking males. Affiliating with high-ranking males could increase an infant’s chance of survival, as infants may secure better protection or support (Huchard et al. 2013). In our study population, previous findings revealed that infants suffer high mortality rates when alpha male positions were changed, following the take-over by a newcomer male (Kerhoas et al. 2014). Securing support from a strong ally is the best survival strategy an infant has at its disposal in an infanticidal species (van Schaik and Janson 2000) as the crested macaque seems to be (Kerhoas et al. 2014). Thus, infant crested macaques seem to be under selective pressure to develop bonds with adult males that may provide sufficient protection if an infanticidal male attacks them. Alternatively, infants are usually in the center of the group, and may interact more with high-ranking males simply because they are also more often in the center of the group (Janson 1990; Hemelrijk 2000).

In addition to male rank, affiliation initiated by infants was also influenced by mother-male bond. Mother-male friendship has been found to promote male-infant bonds in chacma baboons (Moscovice et al. 2009) and Assamese macaques (Ostner et al. 2013). This potentially also influences infants’ survival probability as male friends have been found to react more to playback of infanticidal male threats than control males in chacma baboons, a species with high risk of male infanticide (Palombit 2000). In addition, male caretakers also reacted more intensely to the screams of their juvenile associates than to the screams of other juveniles (Moscovice et al. 2009). Mothers and infants may find a male friend, even of low rank, a more reliable protector compared to a high-ranking male who is more likely to invest in future mating and consort. In fact, a consorting male often spends more time in the periphery of the group and far from the proximity of infants (Macaca Nigra Project unpubl. data). However, these infant-initiated affiliations towards adult males may simply be a by-product of mothers’ relationships with these males. In fact, in chacma baboons, mothers are usually responsible for these male-female partnerships (Palombit et al. 1997; Huchard et al. 2010). If this is also the case in crested macaques, infants may merely copy their mother’s preference for specific interaction partners.

We also found that infants are more likely to affiliate with males when the mother is absent. This might, in the first place, appear contradictory given that crested macaque mothers are considered to have a high degree of permissiveness when group members interact with their infants (Thierry et al. 2000b). Therefore, the presence of the mother should not impede the infant’s affiliations towards males. Several reasons may explain our finding. First, the mother is the primary bonding partner for an infant (Broad et al. 2006), and thus when the mother is absent, the infant will have more occasions to engage with other group members, particularly a close-by male friend of the mother. Second, infants’ motor skills increase with age (included here as a control variable), along with the steady decrease of the mother’s presence in the infant’s proximity. Thus, infants’ affiliations with adult males, when the mother is not present, might be a by-product of their increased motor independence. Third, when mothers are absent, infants may alternatively seek the proximity of an adult male for potential protection (Borries et al. 1999) or greater access to resources (Huchard et al. 2013).

Surprisingly, and in contrast to our prediction, paternity had no effect on infants’ affiliations directed towards adult males. Previous studies discussed several explanations for the absence of kin effects (e.g., *G. beringei beringei*, Rosenbaum et al. 2015): either the infants do not recognize their father, or infants recognize their father but do not benefit from affiliating with him (cf. Mateo 2002). In fact, it has been proposed that the display of a strong father-offspring relationship or phenotypic similarity may prompt infanticide attacks from other males in the group (Johnstone 1997; Pagel 1997; Mateo 2002). However, the fact that infants prefer to affiliate with high-ranking males or mother’s low-ranking friends, especially in mother’s absence, suggests that infants are under pressure to secure their survival by bonding with a strong or a close-by protector, regardless of whether they are the father or not. As an alternative, but mutually not exclusive, explanation, our finding may indicate that infants try to affiliate with the most likely father or at least with an individual who most likely assumes to be their father (as proposed in Rosenbaum et al. 2015). Nevertheless, the mismatch in paternity by both offspring and fathers remains to be addressed in more detail in future research.

### Male model

In contrast to infants, adult males’ affiliative behavior towards infants did not decrease but increased in the presence of the mother. In addition, males in a strong affiliative relationship with the mother affiliated more often with her infant than other infants. Both of these results provide support for the assumption that crested macaque males may use the “care-then-mate” strategy (Ménard et al. 2001) to increase their future reproductive success. In fact, if adult males try to increase their future reproductive success through caring for an unrelated infant, we expect to observe an increase in male affiliations towards an infant when its mother is present (Ménard et al. 2001; this study) as well as an increase in affiliations between the male and the mother (Smuts 1985, this study). However, it remains unclear from the available data of this study whether the unrelated caretakers actually had an increase of future mating and reproductive success with the mother. Hence, future studies including data on the consecutive mating and breeding success are needed to confirm this strategy in more detail. As an alternative, male affiliative behaviors towards infants, consisting mainly of socio-positive approaches, may be a by-product of the males approaching and affiliating with the mother, rather than targeting the infant, as mothers may represent a more valuable social partner for males. In fact, given that infants are not often in proximity of their mother, the results that male affiliate with infants more when the mother is present seems to corroborate that males may target mothers rather than their infants. Thus future studies should investigate male affiliations towards lactating mother with either a dependent or independent infant, in order to disentangle whether the males direct their affiliations towards the infant or its mother.

Our results also stress that high-ranking males were more likely to initiate affiliations with infants, regardless of the mother presence. High-ranking males in crested macaques may interact more with infants as they interact in general more with conspecifics and have more group members in close spatial proximity (Reed et al. 1997). In addition, they may interact more with infants due to their higher paternity likelihood. Interestingly, our results show that adult males present at conception interacted more with an infant than males that were not present, suggesting that males may use this cue to assess their paternity probability (cf. “hedging their bets,” Moscovice et al. 2010). However, in contrast to our prediction, sires in this study do not affiliate more with their offspring. This is surprising, given that preliminary data suggested that paternity certainty might be high in this species, as high-ranking males are potentially able to monopolize females during the fertile phase of their conceptive cycles (AE unpubl. data). As all but one father (99.7 %) were living in the group at the time of birth and as the majority of fathers (86.7 %) were still present when the offspring completed their first year of life, it is unlikely that mismatches in father-offspring affiliation are due to absence of genetic fathers (cf. Moscovice et al. 2009). Fathers were even of similar dominance rank compared to the time of conception (Macaca Nigra Project unpubl. data) which should ensure that male access to infants was not reduced. It is more likely that high-ranking fathers may not spend time caring for their own offspring, but rather trying to increase their mating success, thus maximizing the number of future offspring rather than the survival of present offspring. This should be particularly relevant in this reproductively aseasonal species where there is always at least one cycling female and male tenure can be particularly short (Marty et al. 2015). Shorter alpha male tenure in crested macaques may in fact provoke the lack of paternal care, and it is likely that males, independent of paternity, display low levels of investment as they may not be present to provide long-term care in this dynamic social system.

Male care in primates has been found to range from low-cost care (e.g., savannah baboons, when fathers side with their own offspring involved in conflicts with another juveniles, Buchan et al. 2003) to high-cost care (e.g., Hanuman langurs, when males protect infants against infanticidal males, Borries et al. 1999). Our results show that, in crested macaques, there may only be low-cost care for infants, generally not provided by the father, but instead by unrelated males. Future studies may investigate the level and strength of investment that males dedicate to infants in primate species. In addition, future studies could specifically investigate the influence of different types of affiliative behavior on males and infants bonds.

Mother rank did not influence male-infant affiliations, since neither infants from high-ranking mothers were more likely to initiate affiliations with adult males, nor did adult males prefer to affiliate with them. This is contrary to other studies that found a positive influence of maternal rank on male-infant affiliations in cercopithecines (Huchard et al. 2013; Langos et al. 2013), but in agreement with a previous study in this population showing no maternal rank effect on infant survival (Kerhoas et al. 2014).

## Conclusions

In conclusion, infants affiliate mainly with a male closely bonded with their mothers or a high-ranking male to possibly increase male support during agonistic encounters. Adult male crested macaques did not seem to provide more care to their own offspring than to unrelated infants; however, they probably provided infant care to secure future matings. Females mate promiscuously to confuse paternity, presumably to avoid infanticide, but this may also mean that their infants do not receive protection from the father. This may be the reason why infant mortality is rather high in crested macaques (Kerhoas et al. 2014). Overall, our study revealed for the first time that crested macaques form bonds between adult males and lactating females, which influence the behavior of infants. Future studies should investigate the strength of the mother-male bond and its influence on infant survival. Furthermore, more data are needed to understand whether males who develop a strong bond with a female are more likely to be the father of subsequent infants of this female. Infants have been labeled as “commodities” in social interactions (Henzi and Barrett 2002); however, we observed that infants initiated two thirds of all adult male-infant affiliations, suggesting that infants are the driving force behind adult male-infant relationships and should be the focus of future studies on adult male-infant interactions.

## Electronic supplementary material

Below is the link to the electronic supplementary material.

ESM 1 (DOCX 60 kb)
