# Supplementary material for: Mother-male bond, but not paternity, influences male-infant affiliation in wild crested macaques
Source: Behav Ecol Sociobiol. 2016 Apr 28;70:1117–30. doi: 10.1007/s00265-016-2116-0 (PMC4954837; doi:10.1007/s00265-016-2116-0)
Supplement: Supplementary file 1 — (DOCX 60 kb) [file 265_2016_2116_MOESM1_ESM.docx]

**Supplementary Material:**

Mother-male bond, but not paternity, influences male-infant affiliation in wild crested macaques

Behavioral Ecology and Sociobiology

Kerhoas D, Kulik L, Perwitasari-Farajallah D, Agil M, Engelhardt A and Widdig A

Corresponding author: Daphne Kerhoas, Bristol Zoological Society, [dkerhoas@bristolzoo.org.uk](mailto:dkerhoas@bristolzoo.org.uk)

Table S1. Variables included in the two GLMM analyses on male-infant affiliations.

**Variables**

**Model tested**

**Variable type:**

**Description (all parameters were calculated daily)**

Response variables:

Infant affiliations towards a male

Infant

categorical (0/1)

Male affiliations towards an infant

Male

categorical (0/1)

Predicting variables:

Paternity

Infant & Male

categorical (yes/no)

Based on genetic analysis

Male rank

Infant & Male

continuous (0 to 1.0)

Elo ratings of adult males' hierarchy (rating of 1 is the highest)

Male presence at conception

Male

categorical (yes/no)

Male presence in the group during the swelling period of the infant's

conception

Mother and male friendship

Infant & Male

continuous (0 to 0.17)

Proportion of affiliations when both the male and the mother

were in the infant's proximity

Mother rank

Infant & Male

continuous (0 to 1.0)

Elo ratings of adult females' hierarchy (rating of 1 is the highest)

Mother presence

Infant & Male

discrete (0-96)

Number of scans when the mother is in proximity of her infant

Control variables:

Number of males

Infant & Male

discrete (3-17)

Number of adult males in the group

Infant gender

Infant & Male

categorical (female/male)

Infant age

Infant & Male

discrete (0-366)

infant age in days

Seasonality cosine

Infant & Male

continuous (-1.0 to 1.0)

cosine of date to account for food quantity and availability

Seasonality sine

Infant & Male

continuous (-1.0 to 1.0)

sine of date to account for food quantity and availability

Infant autocorrelation terms

Infant & Male

fixed

to account for temporal interdependence of the response

Male autocorrelation terms

Infant & Male

fixed

to account for temporal interdependence of the response

Male tenure

¹

Infant & Male

discrete (1-1549)

number of days since the beginning of the study

Offset variable:

Observation time

Infant & Male

discrete (1-128)

Number of scans recorded on focal infants by day

Random variables:

Group identity

Infant & Male

categorical (R1/R2/PB)

name of the group of the focal infant

Male identity

Infant & Male

categorical (total of 27)

name of the males present in the group on a given day

Infant identity

Infant & Male

categorical (total of 30)

name of the infants present in the group on a given day

Dyad identity

Infant & Male

categorical (total of 217)

name of both males and infants

Day

Infant & Male

categorical (total of 452)

name of the days of observation

Interactions:

Paternity X Mother presence

Infant & Male

Male rank X Mother presence

Infant & Male

Mother rank X Mother presence

Infant

Infant gender X Mother presence

Infant

¹ Originally male tenure was not in the model, but when plotting we discovered that male tenure was an important component.

Random slopes

We included several random slopes in the model, i.e. for each random effect we considered the following the random slopes term considered: Within group identity, we considered paternity, male rank, mother rank, mother presence, mother-male affiliations, gender, two-way interaction Male rank*Mother presence, two-way interaction Paternity*Mother presence, two-way interaction Mother-male affiliation, three-way Male rank*Paternity*Mother presence. For infant identity, we considered male rank, mother presence, mother-male affiliations, two-way interaction Male rank*Mother presence. For male identity, we considered only mother presence.

Table S2. Results of GLMM analyses of the infant model: the significant factors influencing the affiliations of infants toward males are marked in bold, including the control variables marked in italics (values not shown for the variables comprised by a significant higher interaction)

| Fixed effects: | Estimate | SE | Df | LRT **χ** ^2^ | P of LRT |
| --- | --- | --- | --- | --- | --- |
| (Intercept) | -5.32 | 0.2 |  |  |  |
| Infant AC term | 0.4 | 0.02 | 1 | 442.66 | <0.001 |
| Male AC term | 0.23 | 0.02 | 1 | 140.53 | <0.001 |
| Paternity | 0.13 | 0.12 | 1 | 1.11 | 0.292 |
| Mother rank^(1)^ | 0.03 | 0.02 | 1 | 1.56 | 0.212 |
| **Mother presence** | -0.17 | 0.05 | **1** | **5.27** | **<0.022** |
| **Male rank* Mother-male affiliation** | -0.07 | 0.03 | **1** | **4.36** | **0.037** |
| *Infant gender* | -0.02 | 0.05 | 1 | 0.12 | 0.734 |
| *Number of males* | 0.01 | 0.03 | 1 | 0.06 | 0.811 |
| ***Infant age*** | 0.46 | 0.03 | **1** | **169.72** | **<0.001** |
| ***Male tenure*** | 0.34 | 0.06 | **1** | **25.91** | **<0.001** |
| ***cosine(day)*** | 0.11 | 0.03 | 2 | 15.31 | **<0.001** |
| ***sine(day)*** | -0.09 | 0.04 |  |  | **<0.001** |

^(1)^: larger values indicate larger rank

Table S3. Results of GLMM analyses of the male model: the significant factors influencing the affiliations of males toward infants are marked in bold, including the control variables in italics.

| Fixed effects: | Estimate | | SE | Df | LRT **χ** ^2^ | P |
| --- | --- | --- | --- | --- | --- | --- |
| (Intercept) | | -5.47 | 0.18 |  |  |  |
| Infant AC term | | 0.18 | 0.02 | 1 | 89.27 | <0.001 |
| Male AC term | | 0.19 | 0.02 | 1 | 90.01 | <0.001 |
| Paternity | | -0.08 | 0.1 | 1 | 0.53 | 0.465 |
| Mother rank^(1)^ | | -0.01 | 0.02 | 1 | 0.15 | 0.696 |
| **Mother presence** | | 0.1 | 0.03 | **1** | **4.43** | **0.035** |
| **Male rank**^(1)^ | | 0.14 | 0.06 | **1** | **3.15** | **0.015** |
| **Male presence at conception** | | 0.35 | 0.14 | **1** | **6.02** | **0.014** |
| **Mother-male affiliation** | | 0.07 | 0.03 | **1** | **5.01** | **0.025** |
| *Infant gender* | | 0.03 | 0.05 | 1 | 0.34 | 0.559 |
| ***Numbers of males*** | | -0.09 | 0.03 | **1** | **5.90** | **0.015** |
| *Infant age* | | -0.03 | 0.03 | 1 | 1.44 | 0.230 |
| ***Male tenure*** | | 0.25 | 0.05 | **1** | **17.58** | **<0.001** |
| *Cosine day* | | -0.02 | 0.04 | 2 | 0.21 | 0.899 |
| *Sine day* | | 0.00 | 0.04 |  |  |  |

^(1)^: larger values indicate larger rank
